# Delayed diagnosis of oral squamous cell carcinoma: a case series

**DOI:** 10.1186/1752-1947-5-291

**Published:** 2011-07-06

**Authors:** Nihat Akbulut, Bengi Oztas, Sebnem Kursun, Sehrazat Evirgen

**Affiliations:** 1 Ankara University Dentistry Faculty, Department of Dentomaxillofacial Surgery, Besevler, Ankara, Turkey; 2 Ankara University Dentistry Faculty, Department of Dentomaxillofacial Radiology, Besevler, Ankara, Turkey; 3 Başkent University, Dentistry Faculty, Department of Dentomaxillofacial Radiology, Anıttepe, Ankara, Turkey

## Abstract

**Introduction:**

In malign neoplasms, oral cancer is one of the important causes of mortality and morbidity. Squamous cell carcinoma is the most common form of oral cancers in adults and is related to risk factors such as smoking and alcohol consumption.

**Case presentation:**

In this article, we present three case reports of oral squamous cell carcinomas with delayed diagnosis. The first patient was a 52-year-old Turkish man, the second patient was a 61-year-old Turkish man and the third patient was a 60-year-old Turkish woman. All were referred to the Ankara University Faculty of Dentistry with pain, swelling and various complaints in their jaws.

**Conclusion:**

Early diagnosis is of vital importance for the prognosis of the patients with oral squamous cell carcinomas. For this reason, dentists play a crucial role in the early detection and prevention of oral cancers.

## Introduction

Oral squamous cell carcinoma (OSCC) accounts for approximately 3% of all malignancies and more than 90% of cancers of the oral cavity and oropharynx [1,2]. The reported etiological agents and risk factors for oral cancer include tobacco use, frequent alcohol consumption, the use of areca nut, a compromised immune system and a history of dietary habits that can cause cancer, as well as less established factors such as infection with certain types of human papillomaviruses [3]. OSCC mostly affects adult men between the sixth and seventh decades of life [4,5]. The most affected sites, in decreasing order, are the tongue, oropharynx, lips, floor of the mouth, gingiva, hard palate and buccal mucosa [4]. Clinical stages (tumor, node and metastasis, or TNM) of OSCCs at diagnosis have an important influence on the survival and prognosis of patients. Unfortunately, approximately 60% to 65% of oral cancer patients are in TNM stages III and IV. Delay in diagnosis consists of either patient delay or professional delay [5]. The treatment of choice for OSCCs is wide *en bloc *excision of the tumor in the soft tissue, with the involved bone and post-operative radiotherapy depending on the final histopathological results [6]. Three patients with OSCCs, along with the clinical and radiological findings, are described in this case presentation.

## Case presentation

### Case 1

**Figure 1:**
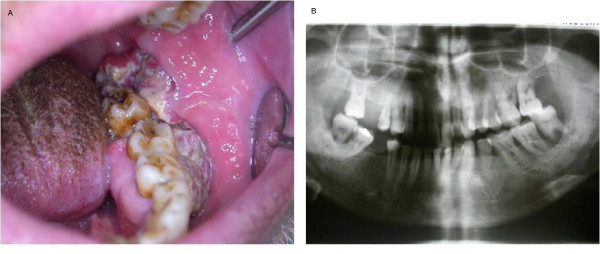
****(a) **Intra-oral view showing the clinical aspect of case 1**. **(b) **Panoramic radiograph showing bone loss in the left mandibular third molar region of case 1.

A 52-year-old Turkish man was referred to the Ankara University Faculty of Dentistry with complaints of pain and swelling in his left mandible and difficulty swallowing. The patient's anamnesis revealed that during a six-month period, he had been to two dentists in his own country, and they had implemented palliative treatment such as the use of mouthwash and antibiotic therapy. Because of the late diagnosis, the lesion was fairly enlarged when he presented to our faculty. His medical history revealed that he had no systemic health problems but had been a smoker for 30 years. His clinical examination revealed a hyperemic, ulcerated area with exophytic enlargement to the buccal and lingual sides (Figure 1a). Because of his lack of oral hygiene, food debris had accumulated on the surface of the lesion and his tongue was hairy. He had an enlarged, painless, fixed lymph node in the submandibular area. A panoramic radiological evaluation revealed a radiolucent area and bone loss with irregular borders in the third molar region of the left mandibula (Figure 1b). Upon an intra-oral and radiological examination, we observed a lesion approximately 6 cm in size. The diagnosis after histological examination of the biopsy specimen was invasive squamous cell carcinoma, and, based on the histopathological evaluation, the lesion was deemed to be at grade 3 level. On the basis of our clinical, radiological and histopathological evaluations, we made the diagnosis of a stage III lesion. The patient was referred to an oncology hospital. According to recently received information, the patient has undergone chemotherapy after surgery and is now in the control period.

### Case 2

**Figure 2:**
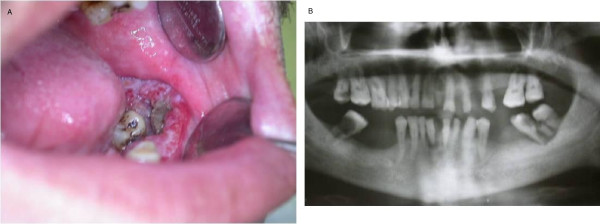
****(a) **Intra-oral view showing the clinical aspect of case 2**. **(b) **Panoramic radiograph showing bone loss in the left mandibular molar region of case 2.

A 61-year-old Turkish man was referred to the Ankara University Faculty of Dentistry with complaints of pain and swelling in his left mandible. His medical history revealed that he had no systemic disorder. He did not have any habits such as smoking or alcohol intake. The patient's anamnesis showed that he refused to be examined for nine months. His clinical examination revealed an ulcerated, hyperemic enlargement with an irregular surface in the left retromolar region (Figure 2a). He had no sign of the lymph node in the submandibular area. The patient's radiographic examination showed excessive bone loss, including the second and third molar teeth in the left mandible, with irregular borders (Figure 1b). Upon clinical and radiological examinations, the lesion size was determined to be approximately 5 cm to 6 cm. The diagnosis rendered after the histological examination of the biopsy specimen was a well-differentiated squamous cell carcinoma at the grade 2 level. On the basis of the clinical, radiological and histological evaluations of the lesion, it was deemed to be in stage II. The patient was referred to an oncology hospital. According to recently received information, the patient underwent surgery and was awaiting a decision regarding the initiation of radiotherapy.

### Case 3

**Figure 3:**
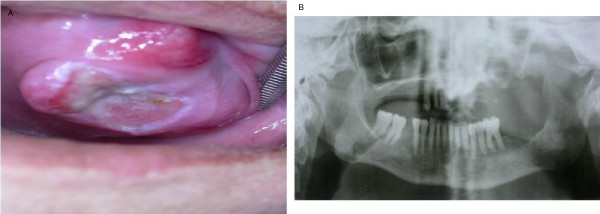
****(a) **Intra-oral view showing the clinical aspect of case 3**. **(b) **Panoramic radiograph showing the destruction of bone with extensive indefinite borders in the maxillary molar region of case 3.

A 60-year-old Turkish woman was referred to the Ankara University Faculty of Dentistry with a complaint of continuous pain for three months after tooth extraction in her maxillary palatal region. During this time period, she had used antibiotics given by her dentist. Her medical history revealed that she had hypertension and diabetes mellitus. She did not have any habits such as smoking or alcohol intake. Her clinical examination showed swelling in her palate with an ulcer in the center of the lesion and a pseudomembrane on it (Figure 3a). Also, she had halitosis. The patient had an enlarged, painless, fixed lymph node in the submandibular area. A radiological examination revealed a considerable expansive area in which the margins of the radiolucent lesion could not be visualized on the panoramic radiograph (Figure 3b). Upon clinical and radiological examinations, the lesion size was determined to be approximately 7 cm. The diagnosis rendered after histological examination of the biopsy specimen was invasive squamous cell carcinoma within the grade 3 level. On the basis of our clinical, radiological and histological evaluations, we determined that the lesion was in stage III. The patient was referred to an oncology hospital.

After undergoing surgery, she was treated with radiotherapy and chemotherapy.

## Discussion

Oral cancer is an important health problem worldwide. According to the World Health Organization, oral cancers have increased in the past few decades [7,8]. The major risk factors for OSCC are smoking and alcohol consumption. The other etiologic factors are genetic pre-disposition, viral pathogens and nutritional habits [7,9,10]. Data published in several reports have indicated that the exposure of women to both tobacco and alcohol causes a change in the susceptibility to oral tumors from a larger ratio of men to women to a larger ratio of women to men [9-11]. In two patients in the present series, one of whom was a woman, their oral tumors were not associated with any habits such as tobacco smoking or alcohol consumption, so their presentations may be attributed to other etiological factors of OSCCs, such as certain viruses (such as human papillomavirus), low consumption of fruits and vegetables, genetic pre-disposition and so forth [10,11].

The survival ratio of patients with head and neck cancers is 76% in cases of early diagnosis without metastasis, 41% in cases involving cervical lymph node metastases and 9% if there is metastasis under the neck region [7]. Dysplastic oral mucosal lesions may develop into OSCCs without early diagnosis and treatment. The survival duration of patients with OSCCs may be lengthened to five years in stages I and II compared with stages III and IV. Patients in stages III and IV are reported to have a mean six months or maximum one year survival duration [8].

Posterior localized tumors demonstrate a worse prognosis, since these often remain unnoticed in screening examinations, and once symptoms arise from regional lymph node metastases, the tumors are at an advanced stage at the time of initial diagnosis [12]. An early diagnosis is not necessarily easy, because patients and health care professionals underrate the initial lesions, which are generally asymptomatic. This reality suggests that physicians have gaps in their knowledge of pathology, that patients delay seeking medical care and that access to and the quality of medical care are deficient, all of which reflect the absence of preventive public health programs and an effective health care system [8-10,13]. All of our patients' diagnoses in this report were delayed. The patients in the present study were referred to an oncology hospital because of their metastases.

In patients with head and neck cancer (HNC) and OSCC, delays in diagnosis of more than one month may contribute to an increased chance of the diagnosis of later-stage disease [14]. Furthermore, Fortin and colleagues [14] found that treatment delays of more than 40 days in early-stage HNC were associated with an increased risk of locoregional failure and an effect on survival. These authors recommended that patients with HNC should be treated within 30 days of diagnosis to achieve improved outcomes [14]. The results of a survey of North American radiation oncologists showed a consensus that delays in initiating radiation therapy of approximately one month from the time of referral were excessive and likely to affect patient outcomes [15].

OSCC and its treatment directly affect patients' health-related quality of life. The most basic functions of speech, chewing and swallowing are frequently altered, while symptoms such as pain and psychosocial issues like appearance and emotional functioning can also be problematic. If these tumors are at an advanced stage, aggressive therapy, including surgery, radiotherapy and, if needed, chemotherapy may be used to treat patients with the worst prognoses [15].

## Conclusion

In terms of quality of life, survival probability and treatment of the patient, early diagnosis of OSCC is very important. Dentists should have enough knowledge about clinical and radiological forms of anatomic structures to diagnose cancer in the oral region. Also, dentists should not overlook any abnormality in the oral region. One of the most important duties of a dentist is good follow-up of patients, especially in the diagnosis period. If indicated, dentists should request a biopsy, and in the presence of metastasis, the patient should be directed to the appropriate related department.

## Consent

Written informed consent was obtained from all three patients for publication of this case report and any accompanying images. Copies of the written consent forms from all three patients are available for review by the Editor-in-Chief of this journal.

## Competing interests

The authors declare that they have no competing interests.

## Authors' contributions

SK wrote the article. BO contributed to writing the manuscript. NA researched and retrieved the references cited. SE was the language supervisor. All authors contributed to this article. All authors read and approved the final manuscript.
